# Identification of cross‐diagnostic protein signatures and subtypes of neurodegenerative diseases using multi‐task deep learning on the UK Biobank proteomics dataset

**DOI:** 10.1002/alz.090583

**Published:** 2025-01-09

**Authors:** Tomasz Gargas, Liping Hou, Tomasz Wrzesinski, Konrad Wojtowicz, Marta Kowalska, Malgorzata Kamuda, Tomasz Jetka, Christopher D Whelan, Bart Smets

**Affiliations:** ^1^ Ardigen, Krakow Poland; ^2^ Janssen Research & Development, LLC, a Johnson & Johnson company, Spring House, PA USA; ^3^ Janssen Research & Development, LLC, a Johnson & Johnson company, Boston, MA USA; ^4^ Janssen Research & Development, A Division of Janssen Pharmaceutica, Neuroscience Therapeutic Area, Beerse Belgium

## Abstract

**Background:**

Neurodegenerative diseases are a heterogeneous group of illnesses. Differences across patients exist in the underlying biological drivers of disease. Furthermore, cross‐diagnostic disease mechanisms exist, and different pathologies often co‐occur in the brain. Clinical symptoms fail to capture this heterogeneity. Molecular biomarker‐driven approaches are needed to improve patient identification & stratification with the aim of moving towards more targeted patient treatment strategies.

**Method:**

The UK Biobank Pharma Proteomics Project has generated a proteomics dataset of unprecedented size. It consists of plasma proteomic profiles measured using the Olink 3k protein panel from over 54,000 individuals, complemented with broad phenotypic & genetic information. It is a unique resource to explore the biological correlation between neurodegenerative diseases as well as with other indications. We applied a multi‐task deep learning (MTL) approach to generate models that predict disease status based on plasma proteomics profiles for a broad range of indications, including neurodegenerative diseases such as Alzheimer’s and Parkinson’s disease. The MTL approach first learns fundamental biological patterns by knowledge sharing between diseases in the initial segment of the network. Subsequently, understanding of a specific indication is refined with dedicated training. This improves model generalizability and statistical power. The neural network also produces low‐dimensional embeddings of proteomic profiles that can be used for sample clustering and to derive insights about disease‐associated processes.

**Result:**

The average precision‐recall area‐under‐the‐curve (PR‐AUC) of the MTL models across all diseases is 0.72 vs. 0.67 for the baseline single task logistic regression models. For Alzheimer’s disease, the MTL classifier has a PR‐AUC of 0.76. Feature importance scores were calculated using the SHAP method. Top features for Alzheimer’s disease included several known biomarkers (e.g., GFAP, NPTXR). A UMAP projection of all diseases using the feature importance scores clusters diseases by disease category. Sample clustering revealed biologically interpretable patient subgroups, such as a Parkinson’s cluster linked to lysosomal biology.

**Conclusion:**

High performance of the MTL approach signifies good characterization of cross‐disease biology. This is corroborated by the model’s capability to produce meaningful low‐dimensional representations of plasma proteomics profiles that can be used for identification of cross‐diagnostic protein signatures and subtypes of neurodegenerative diseases.

UKB application number 65851.

